# Immune Check Point Inhibitors in Primary Cutaneous T-Cell Lymphomas: Biologic Rationale, Clinical Results and Future Perspectives

**DOI:** 10.3389/fonc.2021.733770

**Published:** 2021-08-16

**Authors:** Gabriele Roccuzzo, Silvia Giordano, Paolo Fava, Alessandro Pileri, Alba Guglielmo, Luca Tonella, Martina Sanlorenzo, Simone Ribero, Maria Teresa Fierro, Pietro Quaglino

**Affiliations:** ^1^Department of Medical Sciences, Section of Dermatology, University of Turin, Turin, Italy; ^2^Dermatology-IRCCS Policlinico di Sant’Orsola Department of Experimental, Diagnostic and Specialty Medicine, Alma Mater Studiorum, University of Bologna, Bologna, Italy; ^3^Dermatology Unit, Department of Experimental, Diagnostic and Specialty Medicine, University of Bologna, Bologna, Italy; ^4^Department of Medicine, Institute of Cancer Research, Medical University of Vienna, Vienna, Austria

**Keywords:** Cutaneous T-cell lymphomas, immunotherapy, Mycosis fungoides, immune-checkpoint-inhibitors, Sézary syndrome, nivolumab, pembrolizumab

## Abstract

Primary cutaneous T-cell lymphomas (PCTCL) are the most common types of cutaneous lymphomas, with Mycosis fungoides as the most frequent subtype. Besides early stages which usually have a good prognosis, advanced stages remain a great therapeutic challenge with low survival rates. To date, none of the currently available therapeutic options have significantly improved the outcomes of advanced cutaneous lymphomas. Recent studies have demonstrated that immune-checkpoint molecules, such as PD-1 and CTLA-4, play part in the proliferation pathways of neoplastic T-cells, as well as in other tumors. Hence, the potential role of immune-checkpoint-inhibitors in treating cutaneous lymphomas has been investigated in the last years. Herein, we outline the current knowledge regarding the role of immune-checkpoint molecules in PCTCL, their signaling pathways, microenvironment and therapeutic inhibition rationale. Moreover, we review the published data on immunotherapies in PCTCL and summarize the currently ongoing clinical trials in this field.

## Introduction

Primary cutaneous lymphomas (PCL) are a family of rare non-Hodgkin’s lymphomas (NHL) characterized by monoclonal proliferation of malignant lymphocytes in the skin. Among them, 75% are represented by Cutaneous T-cell lymphoma (CTCL), with Mycosis Fungoides (MF) as the most common subtype, while 25% are Cutaneous B-Cell lymphoma (CBCL) (1). Rarer yet more aggressive, defined by the triad of T-cell leukemic evolution, lymphadenopathy and erythroderma, is the Sézary syndrome (SS) form, which can develop as a final manifestation of MF or appear *de novo* (2). Recently it has been suggested that due to their different T-cell subsets origin SS and MF may represent two distinct pathological entities (3). In the recent years, there has been a growing interest in the understanding of molecular and immunological mechanisms that play a role in tumor development and progression: for instance, it has been well documented that the host’s immune system acts as an active player in modulating the defense response against tumor progression (4). These findings have led to an unprecedent development of new immune-based therapies in the oncology field (5). To date, the world of immuno-oncology, which comprehends all those treatments aimed at manipulating the host’s immune system in order to stop tumor proliferation, has achieved remarkable results in several tumors, such as melanoma, lung, kidney, and bladder cancer (6–9). Interestingly, recent studies on the pathogenesis of CTCL have also identified potential immunological targets for therapeutic approaches aimed at enhancing cell-mediated immunity (10). In particular, the role of immune checkpoint antibodies against PD1 (Programmed cell death protein 1) has been subject of inquiry in the last few years, as it has been proved that by targeting inhibitory PD-1 molecules expressed by exhausted T cells, these drugs can revitalize antitumor T cells and lead to impressive clinical responses (11, 12). In this review of the literature, we outline the current evidence on the interactions between CTCL and the immune system, review the published data on immunotherapies for CTCL and summarize the noteworthy ongoing clinical trials in this field.

## Primary Cutaneous T-Cell Lymphomas: An Overview

The cluster of primary cutaneous T-cell lymphomas (PCTCL) encompasses several lymphomatous entities with common defining underlying features (13). Among them, MF is the most common subtype, representing around 55% of the cases, with an incidence rate of about 5.6 per million people and a stable trend in the last two decades (14). Regardless of the traditionally described histopathological variants (i.e., folliculotropic, pagetoid reticulosis, granulomatous slack skin), the current 2007 staging system is based on a tumor-node-metastasis-blood involvement (TNMB) classification and correlates the clinical features with the prognosis (15). Early stages (IA, IB, IIA), characterized by long-standing erythematous scaly patches/plaques, typically located in the bathing trunk areas, usually show an indolent course: even though the 5-year disease free survival is high (i.e., varying from 98% to 89%), there is still considerable morbidity from pain, itching, discomfort, and disfigurement (16–20). Moreover, according to an Italian retrospective study on 1,422 MF patients, 29.7% of early-stage disease develops a disease progression (18). Advanced stages are conversely identified by skin tumors (stage IIB) or erythroderma (stage III), while blood (stage IVA1), nodal (stage IVA2) and visceral involvement (stage IVB) define the most severe extracutaneous forms (16, 19, 21). Regardless of the clinical onset, MF patients can later develop systemic manifestations of SS (22). The survival rates dramatically drop in the most advanced stages, with 5-year OS rates falling from 56% in IIB to 18% in IVB stages (16). Along with the clinical features, other factors contribute to the biological evolution of the disease: for example, age over 60, large-cell transformation and increased LDH values have been described as independent unfavorable variables (23). Still today prompt diagnosis remains a great challenge for clinicians, as almost 9 out of 10 cases show a significant time delay between symptoms onset and confirmed diagnosis (19).

## Current Therapies for CTCL

All the most recent published treatment guidelines agree on a stage-driven strategy, in consideration of clinical presentation, symptom burden and patient’s comorbidities. However, due to the lack of strong evidence from clinical trials, there is currently no unanimous agreement on the sequence by which treatments should be administered: in fact, the choice of any specific therapy should primarily take into account several factors such as disease subtype, patient age/comorbidities, disease extension and treatment availabilities (24). The main goal of therapy is to improve the quality of life, by reducing symptoms, as durable complete remission is rarely achieved (24). With the exception of few selected stage IA patients, in which expectant policy and watchful waiting may be considered, treatment is recommended in all other cases. In early stages (IA, IB, IIA) first line options include skin direct therapies (SDT) such as topical corticosteroids, topical bexarotene, ultraviolet phototherapy, radiation therapy and the recently EMA-approved topical chlormethine (25). Those refractory to the first line may be considered for systemic therapies, such as retinoids, Interferon alpha, Total Skin Electron Beam therapy (TSEB) or low-dose methotrexate, which conversely represent first-line treatments for stage IIB MF (25, 26). Stage III patients may benefit from extracorporeal phototherapy (ECP), whilst refractory and stage IV patients have been traditionally treated with chemotherapy regimens (gemcitabine, pegylated liposomal doxorubicine, CHOP and CHOP-like polychemotherapy) (26). Patients with advanced MF or SS still have an unmet clinical need of effective treatments, due to low response rates, short-lived improvements, concomitant immunosuppression, and often severe drug-related side effects. Overall survival rates in SS are still low, varying from 7.5 to 22.4 months (27). Allogeneic stem cell transplantation (alloSCT), particularly using reduced-intensity conditioning, remains the only treatment option with curative intention for few selected patients (28). Notably, new options have become available in the last years. The anti-CD52 monoclonal antibody Alemtuzumab has shown significant clinical activity in patients with previously treated advanced MF/SS and constitutes a second-line option for patients with advanced disease, although with less efficacy in tumor-stage MF and large cell transformation types (29, 30). The ALCANZA trial led to the approval of the anti-CD30 monoclonal antibody Brentuximab Vedotin in patients with CD30+ MF, showing an ORR lasting at least 4 months of 56% compared to 13% in the control arm in which MTX or bexarotene were administered (31). Moreover, the MAVORIC trial compared the anti-CCR4 antibody Mogamulizumab with Vorinostat, showing a significantly higher ORR in the former arm (28% *vs* 5%) and resulting in mogamulizumab approval for patients with high Sezary cell burden (32). Among histone deacetylase inhibitors, Vorinostat and Romidopsin have been approved by FDA as second line therapies for CTCL patients, whilst they are currently not available in Europe (33, 34). To date, regardless of the encouraging results of some trials on the aforementioned drugs, there is still no curative therapy that has represented a major breakthrough in the outcomes of CTCL. Ultimately, the latest therapeutic frontier has been set in motion by new studies regarding the potential role of immune-checkpoint-inhibitors in CTCL. Fully understanding the tumor microenvironment and its relationship with the host’s immune system is crucial to develop new effective and highly specific immunotherapies.

## The Role of Tumor Microenvironment in Primary Cutaneous Lymphoma

Since the introduction of so-called immunoediting theory an increasing number of studies have focused on the interaction between the malignancy and the microenvironment: non-immune cells (such as antigen-presenting cells), cells exerting immunosuppressive action or activated T-lymphocytes against neoplastic cells (35). All the studies have highlighted such interactions between the tumour and its microenvironment that are fundamental for MF/SS progression. Globally, in the advanced stages there is a switch from an anti-tumour (Th1) phenotype to a tumorigenic (Th2) one. In the early 2000s it was hypothesised that dendritic cells (DCs) may play an important role in CTCL progression (36–42). Indeed, an accumulation of immature DCs has been thought to be related to MF progression, owing to the immune-suppressive actions that immature DCs may play on activated T-cells, leading to anergy. Another debated category of cells are immune-suppressive cells such as T-reg cells or myeloid derived suppressor cells (MDSCs). In pioneering studies the former has been proposed as the normal counterpart of MF cells, evidence confuted later by the introduction of more specific immunohistochemistry antibody (43–50). Today, the role of Tregs seems to be related to a therapeutic response but it is still far to be fully understood (51–53). Studies on MDSCs are few and hypothesise a role in MF progression as well as the fact that MDSCs can be a marker of treatment response (42, 54, 55). Another intriguing category of cells are tumour-infiltrating lymphocytes (TILs). TILs try to control malignant T-lymphocytes and the main problem is that currently no specific markers can be used to distinguish benign from malignant T-cells. In advanced stages it has been proposed that an accumulation of exhausted anti-tumour cells may be one of the events leading to immune-suppression in MF/SS (56). Furthermore, in contrast to the plasticity of malignant T-cells that can express different phenotypes, TILs may have a constrained one (57). Consequently, malignant T-cells may have the ability to elude the control of the immune system. Eosinophils, macrophages, and endothelial cells may play a role in MF/SS progression. In hematologic malignancies it has been proposed that macrophages may recruit eosinophils *via* the production of vascular endothelial growth factors (VEGFs) (58, 59). However, it’s still unclear whether eosinophils may exert an anti-tumour or tumorigenic role (60). Eosinophils within MF/SS infiltrate are rare. Most of the studies on the role of eosinophils provide contrasting results. Indeed, some groups have observed a significantly higher number of eosinophils in the advanced stages, while other studies have not found correlations between the eosinophil level and the disease stage (61–65). Currently, most studies suggest that eosinophils may play a tumorigenic role in MF/SS or may not exert an anti-tumour action at all. The role of macrophages in CTCLs is clearly tumorigenic and mounting evidence has proven that a polarisation to M2 (CD163+) macrophages is related to disease progression. M2 macrophages have an immune suppressive role leading to MF/SS progression. M2 macrophage accumulation starts in early MF phases and increases in the plaque and tumour stages (66–69). Some Authors have observed an accumulation of periostin-stimulated macrophages in plaque-stage MF that may lead to formation of the tumour lesions, while M2 macrophages may play an important role in maintaining an immunosuppressive tumour microenvironment later. Moreover, the interaction between neoplastic T-cells and the microenvironment also involves keratinocytes, fibroblasts and endothelial cells. A loop has been hypothesised between neoplastic elements and keratinocytes as well as fibroblasts that may lead to a permanent activation of STAT proteins with the production of tumorigenic (Th2) molecules (70). STAT overexpression determines a feedback loop between keratinocytes, stromal, and malignant T-cells leading as a consequence to a Th2 polarization of the inflammatory milieu and an empowerment of STAT overexpression (71). Evidence that endothelial cells can play an important role in MF progression has been clearly observed. By comparing MF infiltrate with healthy donor skin different groups have been proven to have an increase in microvascular density in MF (42, 72–75). Moreover, markers of both neo-angiogenesis such as VEGFA or lymph-angiogenesis (VEGF-C) have been observed as overexpressed in MF/SS highlighting the concept that during MF progression both an increase in blood and in lymphatic vessels can be advantageous to tumour survival and spread (42, 73). In conclusion, in CTCL a switch from an anti-tumour to a tumorigenic phenotype can help the disease to survive and spread to the lymph-nodes and the internal organs. Indeed, the accumulation of exhausted anti-tumour T cells and the increase in immuno-suppressive cells may lead to a cascade of events including an empowerment of immune-suppressive cytokine release as well as an increase in neo-angiogenesis which has the consequence of providing an advantage to the disease.

## The PD-1/PDl-1 Axis in Primary Cutaneous Lymphoma

The immunological interactions between Programmed cell death receptor 1 (PD-1) and its ligands (PD-L1 and PD-L2) expressed on cell membranes have been well documented in the scientific literature, as engagement of PD-1 with PD-L1/PD-L2 has shown to prevent T-cell activation and proliferation, weakening immune response (76). These findings have represented a breakthrough in the immuno-oncology field, leading to the understanding that tumor infiltrating T-cells are often functionally impaired due to high expression of PD-1 levels, while malignant cells can escape immune surveillance by expressing PD-L1 (77). Similarly, overexpression of other inhibitory checkpoint receptors, such as the B7-ligand known as CTLA-4 (Cytotoxic T-Lymphocyte Antigen 4), has been proved to lessen immune surveillance in tumors (78). Hence, throughout the years, antibodies targeting PD-1 and CTLA-4 have been developed for treating several tumors, with the aim of restoring PD-1+ T-cell function and eventually halting tumor proliferation (79). In the growing field of immuno-oncology, studies have been carried out in order to achieve a thorough understanding of PD-1 and CTLA-4 expression in cutaneous lymphomas as well (80). The results have been diverse and noteworthy. Firstly, it has been shown that PD-1 and CTLA-4 are expressed by malignant cutaneous T-cells in MF and SS, while PD-L1 levels are high in dendritic cells *émigrés* from the skin but low in T-cells themselves (56, 81, 82). The different proportion of PD-1 expressing T-cells in MF and SS groups, reported as 13% in the former and 89% in the latter, has provided further evidence for considering them as two distinct entities (83). Secondly, PD-1 expression can help differentiate SS patients, in which PD-1 is highly expressed on neoplastic CD4+ cells, from patients affected by other inflammatory dermatoses, in which PD-1 is more often expressed by CD8+ cells (84). Klemke et al. proved that loss of CD7 and increased PD-1 expression in > 50% of the lymphocytic infiltrates discriminates SS from other erythrodermic inflammatory dermatoses (85). Kantekure et al. have also suggested that PD-1 expression seems to increase with lymphoma progression, correlating with an enhanced immunosuppressive microenvironment (10). However, while the progressive nature of immunosuppression in CTCL is well recognized, the mechanisms that underlie the immune impairment remain essentially unknown (10). The major part seems to be played by the interaction between PD-1 and its ligands PD-L1/PD-L2, as it leads to the transduction of a signal which inhibits the T-cell function, attenuating the immune response and the antitumor activity (86). Besides CTCL, this increased PD-1 expression has been also reported in several other models of defective immune function, including chronic viral infections (87–90). Conversely, high number of tumor-infiltrating CD8+ T cells in MF lesions correlates with a more favorable outcome (91). Moreover, the understanding that CTCL cells, as well as other cancer cells, are capable of evading immune surveillance has been documented by detecting a reduced TH1-response and an enhanced TH2-switch in MF lesions (92–94). All these aspects, along with the immunosuppression observed during disease progression and the evidence of common alterations in immune checkpoint related genes, have brought clinicians to theorize a therapeutic role of immune check point inhibitors in treating CTCL (95, 96). Herein we summarize the current available results, as far as anti-PD1 and anti-CTLA4 therapies for CTCL are concerned.

## Results of Clinical Trials

To date only few studies related to safety and efficacy of ICI use in treating CTCL have been published. Two open-label trials have shown some significant results. The former is a phase I study conducted by Lesokhin et al. in which nivolumab, administered at dosage of 1 or 3 mg/kg every 3 weeks, showed a good tolerability profile in 81 patients with hematologic malignancies (97). Specifically, in the T-cell lymphoma subset, thirteen patients were affected by MF, five by PTCL (Peripheral T-cell lymphoma) and five by other T-cell lymphomas. The ORR was 15% in patients with MF and 40% in those with PTCLs. 73% (i.e., 17/23) of these patients experienced some kind of adverse events (AEs), most commonly mild fatigue, rash, and pruritus, while 5 patients experienced ≥ grade 3 reactions. The latter is a phase II study in which 24 patients with pre-treated MF (*n=*9) and SS (*n=*15) received Pembrolizumab 2 mg/kg every 3 weeks for up to 2 years (12). In this case, the ORR was 38%, with two CRs (complete responses) and seven PRs (partial responses). The median response follow-up time was 58 weeks. Four patients discontinued treatment due to immune-related side effects, while 53% of the patients with SS experienced cutaneous flare reactions. This occurrence was found to be associated with high PD-1 expression on Sézary cells. Furthermore, interesting preliminary clinical data were obtained in a phase 1b study in which 12 patients with relapsed/refractory PTCL and CTCL received pembrolizumab in combination with pralatrexate, a dihydrofolate reductase inhibitor, or decitabine, a cytidine analog, or both pralatrexate and decitabine (98). One patient achieved CR, two had PR, one stayed in SD (stable disease) and two experienced PD (progression disease). All responses were seen in the triple combination arm of pembrolizumab, pralatrexate and decitabine. This result suggests that the integration of pembrolizumab on an epigenetic backbone is safe and may improve the outlook in patients with PTCL and CTCL. Attention has also been focused on personalized treatments based on genomic features. A recent study by Beygi et al. hypothesized that genomic alterations of PD-L1, detected through Next Generation Sequencing techniques, may help predict response to PD-1 targeting therapy in CTCLs: in fact, the identification of PD-L1 structural variants (SVs) as potential genomic biomarkers of response to PD-1 axis inhibition proved to be helpful in assessing the response to Pembrolizumab in 3 patients with CTCL (99). However, the authors acknowledged the need of further larger studies in order to fully explore the predictive value of PD-L1 alterations in CTLCs. As for CTLA-4 inhibiting antibodies, current data are even more limited, as its efficacy in CTCL has yet to be determined. To date, only two case reports have showed positive results. In a case report by Bar-Sela, a 44-year-old male with MF and melanoma, exhibited a complete resolution of MF cutaneous lesions after treatment with ipilimumab for advanced melanoma (100). In another case report, Sekulic et al. described the rapid response of a SS patient with a rare gene fusion between the extracellular/transmembrane domain of CTLA-4 (which has a high affinity for binding ligands) and the intracytoplasmic domain of PD-1 (101). Ultimately, combination of ipilimumab with nivolumab has been experienced in T-cell lymphomas. In a phase I study of eleven patients, the efficacy of the combination was not superior to nivolumab monotherapy with an ORR of 9% and only 1 PR observed (102). Altogether, these findings regarding the role of PD-1 axis in CTCLs confirm the great need for further investigations in this field (103). Here we outline a synopsis of the currently published studies and the ongoing clinical trials (103, 104) (**Tables 1**, **2**).

**Table 1:** Summary of the published results from the main studies on immunotherapy in CTCL.

Target	Drug	Study Type	N° of pts	Inclusion	ORR	Disease outcome
**PD-1 (Lesokhin)**	Nivolumab	Phase I open-label dose-escalation, cohort- expansion basket	13	Heavily pretreated MF	15%	Duration of response up to 81 weeks
**PD-1 (Khodadoust)**	Pembrolizumab	Phase II	24	MF/SS patients (23 of 24 with stage IIB to IV) and heavily pretreated	38%	8 durable responses (median DOR not reached > 58 weeks)
**PD-1 (Marchi)**	Pembrolizumab in combination with epigenetic drugs	Phase 1b	12	Relapsed/refractory TCL (5 PCTL, 3 AITL*, 1 ATLL°, 2 MF and 1 SS).	6 out of 12 patients evaluable for response at the time of analysis	*Arm B*: 2/4 (CR, PR)
		Three arms (4 patients per arm):				
		**A**: pembrolizumab + pralatrexate		*Angioimmunoblastic T-cell lymphoma		
		**B:** pembrolizumab + pralatrexate + decitabine		*°Adult-T-cell lymphoma/leukemia*		
		**C:** pembrolizumab + decitabine				
**PD-1 (Beygi)**	Pembrolizumab	Case report on 3 patients	3		***Duration of response:***	Pt.1 SD
		**Pt.1** Pembrolizumab + IFNg 6 cycles, Pembrolizumab alone 36 cycles;		Pt.1 Stage IIB MF	Pt.1 12 weeks (first round), 110 weeks (second round in combination with RT) Pt.2 12 weeks Pt.3 9 weeks	Pt.2 Discontinuation due to immune-related pneumonitis Pt.3 PD
		**Pt.2** Pembrolizumab 2 cycles		Pt.2 Stage IVB MF		
		**Pt.3** Pembrolizumab 6 cycles		Pt.3 Stage IIB MF		
**CTLA-4 (Bar-Sela)**	Ipililumab	Case report	1	Stage IA MF	CR	–
**CTLA-4 (Sekulic)**	Ipililumab	Case report	1	Stage IVA SS	PR 6 weeks	Death 3 months after last dose

ORR, Overall response rate; Pt, patient; Arm A, Arm B, Arm C.

**Table 2:** Summary of the currently ongoing trials on immunotherapy in CTCL.

Study	Type of study	Drug	Inclusion criteria	Start date	Primary completion	Study completion
**NCT03063632**	Phase II	Pembrolizumab + Interferon-gamma	Relapsed-Non respondent (stage IB-IVB) MF, SS and Advanced Synovial Sarcoma	Oct 13, 2017	Apr 8, 2021	Apr 8, 2022
**NCT03278782**	Phase I/II	Pembrolizumab + Romidepsin	Relapsed-refractory- non respondent peripheral T-cell Lymphoma	Nov 14, 2017	Nov 30, 2021	Nov 30, 2021
**NCT02581631**	Phase I/II	Nivolumab + Brentuximab Vedotin	Relapsed-refractory- non respondent NHL CD30+	Dec 18, 2015	Jan 16, 2020	Aug 30, 2021
**NCT02978625**	Phase II	Talimogene Laherparepvec followed by Talimogene Laherparepvec + Nivolumab	Refractory T-cell and NK Cell Lymphomas, Cutaneous SCC, Merkel Cell Carcinoma, and Other Rare Skin Tumors.	Sept 18, 2017	June 1, 2022	June 1, 2022
**NCT03011814**	Phase I/II	Durvalumab as a single agent or with Lenalidomide	Relapsed/refractory PTCL including CTCL	March 8, 2017	June 8, 2022	June 8, 2022
**NCT03357224**	Phase II	Atezolizumab	Relapsed or refractory stage IIb-IV MF-SS	Sept 24, 2018	Sept, 2021	June, 2025

## Discussion and Future Perspectives

The role of immune checkpoint inhibitors in the treatment of CTCL still represents a unique challenge in immuno-oncology, as the exact role of PD-1 and its ligands in tumor microenvironment of patients with CTCL is not fully understood and may differ from other tumors (105–107). This peculiarity is related to the fact that the tumor itself arises from CD4+ T-cells, a population responsible for priming of the cytotoxic response; therefore, it has been speculated that targeting immune checkpoints would have implications on the functionality of both helper and cytotoxic T cells (108). Hence, as for the PD-1 axis, a substantial difference can be noted between solid tumors and CTCL: in the former group, neoplastic cells express PD-L1 which binds to PD-1 on T-cells, inhibiting their activity. Therefore, targeting PD-1 with anti-PD-1 antibodies can prevent this inhibitory interaction, restoring T-cell function. Conversely, the peculiarity of PD-1 expression in CTCL resides in the fact that the proliferating neoplastic itself is a CD4+ T-cell. In this specific case, targeting PD-1 with anti-PD-1 antibodies could have a double effect: on the one hand, this could restore the antineoplastic function of TILs as in solid tumors, while on the other, this could promote the proliferation of the neoplastic T-cell population (109, 110). Several questions have been raised and still need to be answered. O’Malley et al. showed that over 86% of malignant T-cells in patients with CTCL express PD-1, compared with 16% of benign T cells, suggesting that preferential expression of PD-1 by malignant T cells may underlie worsening of clinical disease in a subset of patients treated with PD-1 blockade (111). Saulite et al. also emphasized that blocking PD-1 in SS reduces Th2 phenotype of non-neoplastic T-cell and may paradoxically enhance tumor proliferation (105). Similarly, Sivanand et al. brought attention to the controversy that, if expression of PD-1 on malignant T-cell has an inhibitory function, PD-1 blockade can potentially promote tumor growth (110). Another topic of discussion is the heterogeneity of results as far as PD-1 expression on T-cell in MF and SS is concerned: in fact, while some authors have reported an augmented expression in a substantial proportion of both MF and SS patients, others have described it as more relevant in SS only (111, 112). Moreover, recent studies about neoantigen heterogeneity have emphasized the role of mutational load in CTCL: Iyer et al. have proved that as MF progresses, the tumor accumulates somatic mutations and evolves to produce multiple genetic subclones (113). Sivanand et al. suggested that this process has a double effect, as on one hand it leads to higher neoantigen expression and increased opportunities for the neoplasm to be recognized by the immune system, while on the other the increasing subclonal distribution of neoantigens can direct the immune system to discrete subpopulations of the most immunogenic tumor cells (114). This in turn may shield the less immunogenic subclones from the antitumor attack and limit efficacy of immunotherapy in MF (114, 115). As for the other main actor in the ICI class (i.e., CTLA-4), even less evidence has been found so far: in fact, while Querfeld et al. observed a promising higher expression of CTLA-4 in CTCL, Anzengruber et al. reported no significant differences with healthy controls (116, 117). To date, as the combination of anti-PD-1+anti-CTLA-4 showed no benefit over anti-PD1 alone, there are no current active trials on the efficacy of CTLA-4 in CTCL (118). All evidence considered, it remains challenging to come to a univocal conclusion on the efficacy of ICI in CTCL. New interesting data may come from other less characterized, yet worthy of mention, ICI molecules: for example, FRCL3 (Fc receptor-like 3), TIGIT (T-cell immunoreceptor with Ig and ITIM domains), BTLA (B and T Lymphocyte Associated), ICOS (Inducible T-cell costimulator) and LAG-3 (Lymphocyte-activation gene 3) have been found to be significantly upregulated in CTCL and this finding could represent a new frontier in the research of new target therapies (108, 117, 119, 120). Recently, new findings regarding ICOS expression in CTCL cells seem to provide the preliminary basis for therapeutic trials, as anti-ICOS antibody–drug conjugates proved antitumor potential against CTCL cell lines and patient-derived xenografts (121). Nevertheless, no *in vivo* studies testing the blockage of these molecules have been started so far. Finally, it is worth mentioning that few authors have listed T-cell lymphomas among the potential, yet very rare, immune-related adverse events following ICIs use: for instance, in the 2012-2018 FAERS (Food and Drug Administration Adverse Events Reporting System) pharmacovigilance database a 0.02% incidence of T-cell lymphoma post-ICIs use, with a 17% mortality, was registered (122). It has been speculated that this phenomenon might be associated with rebound overexpression of PD-1 after the treatment, however actual mechanisms remain still unknown and further studies are needed to better characterize this paradoxical occurrence (123, 124). In conclusion, this literature review highlights the potential role of immune-check point inhibitors in CTCL, according to the current available data. Altogether, the outlook of using ICI in this field seems to be less favorable compared to the one observed in other tumors, such as melanoma (6). Carrying out new research, aimed at disentangling the complex relationship between CTCL and the host’s immune system, may hopefully lead to a more detailed understanding of immunological targetable molecules, in order to provide patients with innovative therapeutic chances.

## Author Contributions

PQ, MF, SR, and PF conceived and designed the presented review. GR, SG, AP, and AG wrote the manuscript with input from all authors. MS and LT analyzed the data. PQ, MF, SR, PF, GR, SG, AP, AG, MS, and LT contributed to the implementation of the research. All authors contributed to the article and approved the submitted version.

## Conflict of Interest

The authors declare that the research was conducted in the absence of any commercial or financial relationships that could be construed as a potential conflict of interest.

## Publisher’s Note

All claims expressed in this article are solely those of the authors and do not necessarily represent those of their affiliated organizations, or those of the publisher, the editors and the reviewers. Any product that may be evaluated in this article, or claim that may be made by its manufacturer, is not guaranteed or endorsed by the publisher.
